# Changes in leisure activities of the elderly due to the COVID-19 in Korea

**DOI:** 10.3389/fpubh.2022.966989

**Published:** 2022-08-12

**Authors:** Eui Jae Kim, Seong Man Park, Hyun Wook Kang

**Affiliations:** ^1^Department of Recreation and Leisure Sports, Dankook University, Yongin, South Korea; ^2^College of Liberal Arts, Dankook University, Yongin, South Korea

**Keywords:** COVID-19, post covid, the elderly, leisure activity, physical activity, social activity, national leisure activity survey

## Abstract

Recreational activities such as physical and social activities are key components of a healthy life for the elderly. Since the outbreak of Corona 19, leisure facilities such as indoor sports facilities, religious facilities, and cultural facilities have been closed, and group activities such as volunteer activities and clubs are also being held under control. These measures are expected to bring about many changes in the leisure activities of the elderly. In this study, an empirical analysis was made on how COVID-19 caused changes in leisure activities of the elderly using national statistical data. For the data analysis, raw data of the “2019 National Leisure Activities Survey” and “2020 National Leisure Activities Survey” conducted by the Ministry of Culture, Sports and Tourism were used, and data of a total of 5,069 elderly people were analyzed. As for the analysis method, the changes in leisure activities of the elderly before and after COVID-19 in terms of participation rate were compared and analyzed. In addition, the changes in leisure activities of the elderly in terms of the type of leisure activities were examined before and after COVID-19 as well. As a result of the analysis, the participation rate in sports activities, hobbies and entertainment activities, and leisure activities increased, while the participation rates in culture and art viewing and participation activities, sports viewing activities, tourism activities, and social activities decreased. In particular, it was found that the proportion of the elderly spending leisure time centered on active and social activities decreased, and the proportion of the elderly consuming leisure time centered on passive activities increased. As a result, it was found that the leisure activities of the elderly are changing passively due to COVID-19. Leisure support policies for active leisure activities of the elderly are likely to be necessary.

## Introduction

First reported in Wuhan, China in December 2019, Corona virus (later referred to as Covid-19) quickly spread to neighboring countries and developed into a global epidemic (1). The aftermath of COVID-19 is not diminishing even now while vaccinations are in progress, and it is facing a prolonged phase. Unlike previous infectious diseases, which pass as a single social wave or first wave, Corona 19 is expected to act as a catalyst for social change and lead to another wave (second wave), causing a great social transformation (2). In this regard, efforts to predict and prepare for social changes in the post-corona era are required.

COVID-19 is even more lethal for people over the age of 60. The U.S. Centers for Disease Control and Prevention reports that 8 out of 10 deaths from COVID-19 are from people over the age of 65 (3). In Korea, 92.9% of COVID-19 deaths were people over the age of 60 years old, and the mortality rate of COVID-19 for the elderly over 80 years old reached 25.3%, and thus it was found that the mortality rate increased sharply with increasing age (4).

Given the high infection rate and mortality rate among the elderly, the government recommended that elderly people avoid going out as much as possible and live within their residence, and stopped operating welfare facilities for the elderly, such as senior welfare centers and senior citizens' centers. These measures have brought about significant changes in the daily lives of the elderly. Numerous studies have found that there were decreases in physical activity and increases in sedentary behavior in the elderly after the outbreak of COVID-19 (5–11), and various psychological problems including stress, anxiety, and depression (12, 13). According to the results of studies within the Korean context, the overall risk of developing depression among the elderly after the COVID-19 pandemic has doubled compared to before the pandemic; furthermore, it was reported that even for the elderly who had no history of depression, the risk of developing depression increased 2.4 times compared to before the pandemic (14). In particular, it is revealed that the decrease in social activities is a major factor that increases the risk of depression in the elderly in the pandemic era (14).

Recreational activities such as physical and social activities are very important to the elderly. Physical activity contributes to mental wellbeing (15), particularly as a protective factor against viral infections (16). Nieman and Wentz (17) reviewed the evidence for an immune response to physical activity and found that moderate exercise strengthened the immune system and reduced inflammation. Accordingly, medical researchers and institutions in each country are recommending maintaining a healthy life in the era of COVID-19 according to the rules of physical activity (18). Zhang et al. (19) also point out that participation in leisure activities is protective of decline in cognitive abilities and has diverse benefits in health conditions among the elderly.

Besides physical activity, social activity is also related to the health of older people. Social bonds affect health, and these effects last a lifetime (20). Social activity provides a stress buffering effect, and social integration promotes a positive psychological state, resulting in positive physiological responses (21). In particular, social activities of the elderly are associated with a lower mortality rate compared to other leisure activities (22). In addition, the elderly who participate in some leisure activities are likely to experience less depression based on the results of the longitudinal study (23).

These results indicate the importance of maintaining active leisure activities for the elderly during the period of COVID-19.

It is said that the happiness of the elderly is higher when they engage in active leisure activities rather than passive leisure activities (24), and those who enjoy active leisure activities such as club activities, volunteering, and travel have higher life satisfaction (25). The elderly who engage in physical and social leisure activities rather than simple recreational activities for rest have higher health-related quality of life (HRQoL) (26), and leisure satisfaction among the elderly who actively participate in outdoor activities is relatively high (27).

Meanwhile, in the midst of the COVID-19 situation, the society has been focusing on quarantine and trying to contain the spread of the infection. Leisure facilities such as indoor sports facilities, religious facilities, and cultural facilities are closed under the judgment that they are vulnerable to the spread of infectious diseases, and group activities such as volunteer activities and clubs are also controlled. These government measures have brought about many changes in the leisure activities of the elderly. According to a study observing changes in leisure life after COVID-19, the number of cases of enjoying active leisure activities decreased after COVID-19, and the number of cases of enjoying passive leisure activities increased (4). Leisure activities with a social nature, such as family meetings and religious activities, decreased (28), but online-based leisure activities increased (29, 30). In addition, there have been cases of switching to other types of leisure activities due to the limited leisure environments (9), and changes in the form of existing leisure activities (31) have been observed.

Although many studies have observed changes in leisure activities after COVID-19, most studies have focused on students and general adults, so it is difficult to obtain information on what changes have occurred in leisure activities of the elderly after COVID-19. In addition, there are limitations in generalizing the research results because previous studies analyzed a relatively small number of samples (32). Considering the importance of leisure activities in life in old age, it is believed that examining how COVID-19 has changed the leisure lives of the elderly will have great value and implications from academic and practical aspects.

Therefore, this study intends to empirically analyze the changes in leisure activities of the elderly after COVID-19 by using the raw data of the National Leisure Activities Survey in 2019 and in 2020 conducted by the Ministry of Culture, Sports and Tourism in Korea, which has a relatively large number of samples as national statistical data.

The purpose of this study is to understand the leisure behaviors of the elderly in the pandemic situation by examining the changes in the leisure activities of the elderly due to COVID-19. Ultimately, this study is expected to contribute to preparing timely leisure policies in the with- or post-Corona era.

The main research questions of this study are as follows.

Research Question 1. After the outbreak of COVID-19, what changes are there in the leisure activities of the elderly?

Research question 2. After the outbreak of COVID-19, what changes are there in the leisure patterns of the elderly?

## Research method

### Analysis data

The data analyzed in this study are the “2019 National Leisure Activities Survey” and “2020 National Leisure Activities Survey” announced by the Ministry of Culture, Sports and Tourism. The National Leisure Activities Survey is a state-approved statistics that “analyzes the state of the people's leisure activities, identifies changes in lifestyle and quality of life, and uses them as basic data for establishing related policies” (33, 34). The population of the 2019 National Leisure Activities Survey is 10,060 men and women aged 15 and older in 17 cities and provinces nationwide, and the population of the 2020 National Leisure Activities Survey is 10,088 men and women aged 15 and older in 17 cities and provinces nationwide. Specific information on the analyzed data is as follows.

First of all, concerning the research structure of this study, the “2019 National Leisure Activities Survey” and “2020 National Leisure Activities Survey” were conducted by Ministry of Culture, Sports and Tourism, Republic of Korea, with population aged 15 and over nationwide. The number of valid respondents was 10,060 in 2019 and 10,080 in 2020, respectively. As a survey method, a household visit interview survey in which a professional surveyor visits selected households and fills out the responses to the questionnaire was employed. All the statistics in the surveys were approved by the National Statistics Office (approval number: No. 113014) and open to the public.

With regard to the sampling design of this study, the survey population was defined as household members aged 15 years or older residing in all households in Korea at the time of the survey baseline. The sampling frame was decided based on data from the 2017 Census survey by Statistics Korea. For stratification, a total of 17 cities and provinces are stratified into large cities and rural areas which divided into dong (i.e., towns) and eup/myeon (i.e., villages) reflecting the characteristics of urban and rural areas. As a sampling method, random extraction after allocating the number of households in each province by square root proportional distribution considering the precision and appropriateness of the sample was done based on Stratified Multi-Stage Cluster Sampling.

For weight calculation, final weight was calculated as follow:

Design weight^*^Non-response adjustment coefficient^*^(1/In-household extraction rate)^*^Population information adjusted coefficient.

In this study, male and female elderly people in their 60s or older were selected as the study subjects among the valid respondents, and after weighting, 2,526 people in 2019 and 2,543 people in 2020 total 5,069 people. The demographic characteristics of the study subjects are shown in (Table 1).

**Table 1:** Demographic characteristics of the study subjects.

**Characteristics**	**Division**	**2019** **(*****n*** = **2,526)**		**20202019** **(*****n*** = **2,543)**	
		**Frequency**	**Percentage**	**Frequency**	**Percentage**
Gender	Male	1,131	44.8	1,141	44.9
	Female	1,395	55.2	1,402	55.1
Education	Elementary school	948	37.5	153	6.0
	Middle school	556	22.0	740	29.1
	High school	782	31.0	563	22.1
	University	240	9.5	875	34.4
	Missing value	–	–	212	8.3
Marital status	Unmarried	26	1.0	19	0.7
	Married (with a spouse)	1,684	66.7	1,637	64.4
	Bereavement/Divorce/ETC	816	32.3	885	34.8
	Missing value	–	–	2	0.1
Monthly income	<1 million won	715	28.3	709	27.9
	Between 1 and 2 million won	568	22.5	579	22.8
	Between 2 and 3 million won	478	18.9	447	17.6
	Between 3 and 4 million won	321	12.7	336	13.2
	Between 4 and 5 million won	199	7.9	195	7.7
	Between 5 and 6 million won	131	5.2	117	4.6
	More than 6 million won	114	4.5	160	6.3
Regional scale	Large cities	967	38.3	1,060	41.7
	Medium and small cities	660	26.1	734	28.9
	Small towns and villages (e.g., Eup, Myeon areas)	899	35.6	749	29.5

### Analysis variables

The variable used to analyze the type of leisure activity was the questionnaire “Which leisure activity did you engage in the most during the past year?” The response items consist of 88 items for 2019 and 2020, and the participants were allowed to respond to up to 5 leisure activities from 1st to 5th. In this study, all five responses were used for analysis, and they were reclassified into active leisure, passive leisure, and social leisure according to the existing literature (35) as follows.

First, active leisure refers to cultural and artistic viewing and activities, sports viewing and participation activities, tourism activities, and hobbies, excluding rest activities such as watching TV and taking a nap, or social activities such as meeting friends and social gatherings. Examples include cultural and art viewing activities such as visiting museums, theater performances, and exhibitions; cultural and artistic participation activities such as participating art activities, traditional art activities, and playing musical instruments; sports viewing activities such as direct or indirect viewing of sports events; participation activities in sports such as tennis, golf, and swimming; tourism activities such as camping, overseas traveling, and getting aboard a cruise ship; and hobbies such as life crafts gardening, and collection activity.

Second, passive leisure refers to passive activities such as listening to music, watching TV, taking a nap, listening to the radio, or doing nothing.

Last, social leisure refers to socially-oriented activities such as meeting friends, reunions/social gatherings, and club activities. In this study, other activities such as volunteer activities and religious activities were also classified as social leisure activities according to the leisure activity classification criteria of the National Leisure Activities Survey.

### Analysis method

The detailed analysis method of this study is as follows. First, we looked at the changes in leisure activities of the elderly after COVID-19 in terms of participation rate. The percentages (%) that responded to the most frequently participated leisure activities were presented by grouping them into eight major categories, and the percentage difference (%p) and the rate of change were calculated to find out the change.

**Figure 1:**
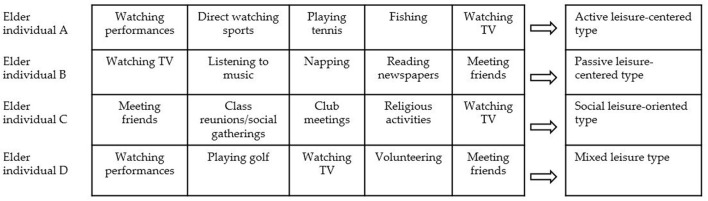
Conceptual diagram of individual leisure activity type.

Next, the changes in leisure activities of the elderly after COVID-19 were examined in terms of the type of leisure activities. By dividing individual leisure activity types into active leisure-oriented type, passive leisure-oriented type, social leisure-oriented type, and leisure mixed type, the percentage (%), percentage difference (%p), and increase/decrease rate before and after COVID-19 were examined. Here, the active leisure-centered type refers to a case in which more than half of the responses to the leisure activities, in which they participated the most, were active leisure. For example, as in the case of elderly person A in Figure 1, individual leisure activities consist of watching performances, direct watching sports, playing tennis, fishing, and watching TV. The passive leisure-centered type refers to a case in which more than half of the responses to an individual's leisure activity type consisted of passive leisure. For example, as in case B in Figure 1, leisure activities consist of watching TV, listening to music, napping, reading newspapers, and meeting friends. The social leisure-oriented type refers to a case in which more than half of the responses to the leisure activities in which they participated the most were composed of social leisure. For example, as in the case of elderly person C in Figure 1, leisure activities consist of meeting friends, class reunions/social gatherings, club meetings, religious activities, and watching TV. Lastly, the mixed leisure type refers to a case in which active, passive, and social leisure is mixed. For example, as in the case of elderly person D in Figure 1, leisure activities consist of watching performances, playing golf, watching TV, volunteering, and meeting friends. Data analysis was performed using IBM SPSS version 23.

## Results

### Changes in leisure activities of the elderly after COVID-19: Aspects of participation rate

The results of analyzing the changes in leisure activities of the elderly after the outbreak of COVID-19 were shown in Table 2. The participation rate of 'sports participation activities, hobbies and entertainment activities, and leisure activities' increased, and the participation rate of ‘culture and art viewing/participation activities, sports viewing activities, tourism activities, and social activities' decreased.

**Table 2:** Changes in leisure activities of the elderly after COVID-19: aspects of participation rate.

**Category**	**Before COVID-19**	**After COVID-19**	**Difference**	
	**A(%)**	**B(%)**	**% Difference B-A**	**Change rate(%) (B-A)/A**
Cultural and art viewing activities	5.5	3.6	−2.0	−35.4
Cultural and artistic participation activities	6.3	5.1	−1.2	−18.9
Sports watching activities	13.3	7.6	−5.7	−42.8
Sports participation activities	18.5	23.6	5.1	27.8
Tourism activities	19.5	12.7	−6.8	−35.0
Hobby and entertainment activities	102.7	104.6	1.9	1.8
Rest activities	221.7	231.7	10.0	4.5
Social and other activities	111.6	102.2	−9.4	−8.5

Specifically, the participation rate of cultural and art viewing activities were 5.5% before COVID-19 and 3.6% after COVID-19, with a percentage difference of−2.0% and a change rate of−35.4%. The participation rate of cultural and artistic participation activities was 6.3% before COVID-19 and 5.1% after COVID-19, with a percentage difference of−1.2% and a change rate of−18.9%. The participation rate of sports watching activities was 13.3% before COVID-19 and 7.6% after COVID-19, with a percentage difference of−5.7% and a change rate of−42.8%. The participation rate of sports participation activities was 18.5% before Corona 19 and 23.6% after Corona 19, with a difference of 5.1% and a change rate of 27.8%. The participation rate of tourism activities was 19.5% before Corona 19 and 12.7% after Corona 19, the percentage difference was−6.8%, and the increase/decrease rate was−35.0%. The participation rate of hobby and entertainment activities was 102.7% before Corona 19 and 104.6% after Corona 19, with a difference of 1.9% and a change rate of 1.8%. The participation rate of rest activities was 221.7% before Corona 19 and 231.7% after Corona 19, with a difference of 10.0% and a change rate of 4.5%. The participation rate in social and other activities was 111.6% before COVID-19 and 102.2% after COVID-19, with a percentage difference of−9.4% and a change rate of -8.5%.

### Changes in leisure activities of the elderly after COVID-19: Aspects of leisure activities

The results of analyzing the changes in leisure activities of the elderly after COVID-19 were explained in Table 3. The active leisure-centered type, with more than half of active leisure activities, showed a decrease of−4.7% from 20.6% before COVID-19 to 19.6% after COVID-19, with a difference of−1.0%p. The passive leisure-centered type, with more than half of passive leisure activities, showed an increase of 20.6%, from 36.9% before COVID-19 to 44.4% after COVID-19, and the percentage difference was 7.6%p. The social leisure-oriented type, in which more than half of the social leisure activities were performed, showed a decrease of−36.6% from 5.0% before COVID-19 to 3.2% after COVID-19, and the percentage difference was−1.8%p. The mixed leisure type, in which active, passive, and social leisure activities are mixed, showed a decrease of−12.7% from 37.5% before Corona 19 to 32.8% after Corona 19, and the percentage difference was -4.8%p.

**Table 3:** Changes in leisure activities of the elderly after COVID-19: aspects of leisure activities.

**Division**	**Before COVID-19**	**After COVID-19**	**Difference**	
	**A (%)**	**B (%)**	**% Difference B-A**	**Change rate (%) (B-A)/A**
Active leisure-centered type	20.6	19.6	−1.0	−4.7
Passive leisure-centered type	36.9	44.4	7.6	20.6
Social leisure-oriented type	5.0	3.2	−1.8	−36.6
Mixed leisure type	37.5	32.8	−4.8	−12.7

## Discussion and conclusion

The purpose of this study is to provide basic data for leisure policy in the post-corona era by empirically analyzing how the leisure life of the elderly has changed since Corona 19. The results derived from this study are discussed as follows.

Since the outbreak of COVID-19, many changes have occurred in the leisure activities of the elderly. Culture, art, sports viewing, tourism, and social activities of the elderly decreased, while sports participation, hobbies, and leisure activities increased. First of all, the most notable result of the leisure activity participation rate is that the rest activity increased. This supports the results of studies that showed that the elderly had decreased physical activity and increased sedentary behavior after the outbreak of COVID-19 (5–7, 9–11). The increase in resting activities, such as watching TV, sleeping, and listening to radio, is not desirable in terms of the health of the elderly, but it may be an unavoidable choice for the elderly who are vulnerable to the virus. Despite the increase in inactive rest activities, the fortunate thing is that sports participation increased. These results are likely to be related to an increase in home-based exercise. The popularity of home training is increasing due to the closure or limited operation of sports facilities, with 8 out of 10 people experiencing home training after the COVID-19 pandemic (36), and the average social postings related to home training in 2020 increased by 112.7% compared to before COVID-19 (37). Hobbies such as gardening, caring for companion animals, and crafts also increased, which can be interpreted as the reason for the increase in hobby activities that the elderly can do alone at home.

On the other hand, in the case of cultural and art viewing activities, there was a decrease. Recently, as the online platform-based non-face-to-face performance culture is spreading, cultural and art viewing activities are becoming more active (38), but this study showed the opposite result. Digitalization is accelerating all over the world due to COVID-19, and this phenomenon is expected to continue even after the end of COVID-19. It seems that policy efforts are needed to ensure that the elderly can equally enjoy the benefits of digital technology without discrimination or exclusion.

There was also a decrease in social activities, and these results are in agreement with the results of previous studies that the frequency of meeting with friends and acquaintances decreased and the leisure time spent alone increased to some extent after Corona 19 (8, 39). In addition, the participation rate of the elderly in tourism activities also decreased, which is in contrast to a recent study that revealed that outdoor and natural activities of adults increased after the onset of COVID-19 (40). These results predict that the older people experience relatively greater restrictions on outdoor activities than the younger ones.

The next notable result in terms of the composition of leisure activities is that the number of elderly people who spend most of their leisure time in passive leisure after the outbreak of COVID-19 is increasing. This is a very unfortunate result considering that the leisure patterns of the elderly in Korea were becoming active and diversified before the outbreak of COVID-19 (35). This result shows the negative changes in the leisure life of the elderly due to COVID-19, and predicts the health problems of the elderly. Inactive leisure activities among the elderly can lead to decreased physical and cognitive functions, decreased muscle mass, and increased levels of inflammation. This can exacerbate chronic diseases and lead to extreme stress and depression, leading to suicide. Therefore, policy measures for the healthy leisure life of the elderly should be prepared.

Combining the above results, this study was able to identify changes in leisure activities of the elderly due to COVID-19. These changes are expected to return to the previous state after the end of COVID-19, but it is expected to continue for a considerable period of time. Based on these analysis results, the researchers intend to draw and present implications that can contribute to timely leisure policies in the post-corona era. First, online recreation services for the elderly need to be activated. At the government level, it is necessary to create an environment where the elderly can receive various recreational services at home by producing various online contents such as sports, art, music, and gardening activities. Furthermore, it is necessary to activate an *Ontact* type of recreational service that enables two-way communication between the elderly and the recreation leader. Second, digital inclusion of the elderly is necessary. First, it is necessary to conduct digital competency education so that the elderly can use online recreation services smoothly. In addition, it is necessary to create an environment where the elderly can freely use the Internet anytime and anywhere, and it is also necessary to consider support for digital devices such as smartphones, tablets, and PCs.

This study is meaningful in that it explains the effect of COVID-19 on leisure activities of the elderly by using national statistical data with relatively high reliability of the survey and a large number of samples. It is hoped that this study will be used as a reference for those involved in the leisure industry and leisure policymakers preparing for the post-corona era.

Finally, this study suggests the following for further research. First, it is proposed to examine whether changes in leisure activities of the elderly due to COVID-19 differ according to economic status, region of residence, number of household members living together, gender, and age. Second, it is suggested that a qualitative study be conducted for in-depth discussion on changes in leisure activities of the elderly after COVID-19.

## Data availability statement

The original contributions presented in the study are included in the article/supplementary material, further inquiries can be directed to the corresponding authors.

## Author contributions

Conceptualization, validation, investigation, data curation, and writing—original draft preparation: EK and HK. Methodology, formal analysis, and visualization: EK. Writing—review and editing: SP. Supervision and project administration: HK. All authors have read and agreed to the published version of the manuscript.

## Conflict of interest

The authors declare that the research was conducted in the absence of any commercial or financial relationships that could be construed as a potential conflict of interest.

## Publisher's note

All claims expressed in this article are solely those of the authors and do not necessarily represent those of their affiliated organizations, or those of the publisher, the editors and the reviewers. Any product that may be evaluated in this article, or claim that may be made by its manufacturer, is not guaranteed or endorsed by the publisher.
